# TRPC3 positively regulates reactive oxygen species driving maladaptive cardiac remodeling

**DOI:** 10.1038/srep37001

**Published:** 2016-11-11

**Authors:** Naoyuki Kitajima, Takuro Numaga-Tomita, Masahiko Watanabe, Takuya Kuroda, Akiyuki Nishimura, Kei Miyano, Satoshi Yasuda, Koichiro Kuwahara, Yoji Sato, Tomomi Ide, Lutz Birnbaumer, Hideki Sumimoto, Yasuo Mori, Motohiro Nishida

**Affiliations:** 1 Division of Cardiocirculatory Signaling, Okazaki Institute for Integrative Bioscience (National Institute for Physiological Sciences), National Institutes of Natural Sciences, Okazaki, Aichi 444-8787, Japan; 2 Department of Translational Pharmaceutical Sciences, Graduate School of Pharmaceutical Sciences, Kyushu University, Fukuoka 812-8582, Japan; 3 Department of Physiological Sciences, SOKENDAI (School of Life Science, The Graduate University for Advanced Studies), Okazaki, Aichi 444-8787, Japan; 4 Department of Anatomy, Hokkaido University School of Medicine, Sapporo 060-8638, Japan; 5 Division of Cell-Based Therapeutic Products, National Institute of Health Sciences, Setagaya, Tokyo 158-8501, Japan; 6 Department of Biochemistry, Kyushu University Graduate School of Medical Sciences, Fukuoka 812-8582, Japan; 7 Department of Cardiovascular Medicine, Kyoto University Graduate School of Medicine, Kyoto 606-8507, Japan; 8 Department of Cardiovascular Medicine, Graduate School of Medical Sciences, Kyushu University, Fukuoka 812-8582, Japan; 9 Laboratory of Neuroscience, NIEHS, NIH, Research Triangle Park, NC 27709, USA; 10 Institute for Biomedical Research (BIOMED), Catholic University of Argentina, C1107AFF Buenos Aires, Argentina; 11 Department of Synthetic Chemistry and Biological Chemistry, Graduate School of Engineering, Kyoto University, Kyoto 615-8510, Japan; 12 PRESTO, JST, 4-1-8 Honcho, Kawaguchi, Saitama 332-0012, Japan

## Abstract

Reactive oxygen species (ROS) produced by NADPH oxidase 2 (Nox2) function as key mediators of mechanotransduction during both physiological adaptation to mechanical load and maladaptive remodeling of the heart. This is despite low levels of cardiac Nox2 expression. The mechanism underlying the transition from adaptation to maladaptation remains obscure, however. We demonstrate that transient receptor potential canonical 3 (TRPC3), a Ca^2+^-permeable channel, acts as a positive regulator of ROS (PRROS) in cardiomyocytes, and specifically regulates pressure overload-induced maladaptive cardiac remodeling in mice. TRPC3 physically interacts with Nox2 at specific C-terminal sites, thereby protecting Nox2 from proteasome-dependent degradation and amplifying Ca^2+^-dependent Nox2 activation through TRPC3-mediated background Ca^2+^ entry. Nox2 also stabilizes TRPC3 proteins to enhance TRPC3 channel activity. Expression of TRPC3 C-terminal polypeptide abolished TRPC3-regulated ROS production by disrupting TRPC3-Nox2 interaction, without affecting TRPC3-mediated Ca^2+^ influx. The novel TRPC3 function as a PRROS provides a mechanistic explanation for how diastolic Ca^2+^ influx specifically encodes signals to induce ROS-mediated maladaptive remodeling and offers new therapeutic possibilities.

The heart comprises a highly dynamic mechanical environment that flexibly changes its structure and morphology to maintain its mechanical properties. In particular, mechanical stretch during diastolic filling has broad implications for cardiac development and the progression of heart failure. Maladaptive cardiac remodeling, defined by excessive accumulation of extracellular matrix components as well as hypertrophic growth of cardiomyocytes is now attracting attention as a leading cause of death worldwide. Much evidence suggests the involvement of chemical stressors, including transforming growth factor β (TGFβ), connective tissue growth factor (CTGF) and angiotensin (Ang) II, in the progression of fibrosis, but drugs targeting these pathways have shown only limited efficacy in human patients^1^. Because both physical (i.e., mechanical) and chemical (i.e., oxidative) stresses participate in the initiation and progression of heart failure, identification of a common target that drives the maladaptive cardiac remodeling induced by hemodynamic overload will be necessary to establish an innovative therapeutic strategy^2^,^3^.

Nox2 is a microtubule-associated ROS-producing enzyme that acts as a key mediator of mechanotransductive signaling in normal hearts^4^. Transient ROS production induced by mechanical stretch during diastolic filling triggers a burst of Ca^2+^ sparks through oxidative modification-dependent activation of ryanodine receptors. By contrast, persistent Nox2-derived ROS production in response to pressure overload in mice leads to oxidative stress through Nox2-derived ROS-induced ROS release from mitochondria and participates in the transition from cardiac adaptation to maladaptation^5^,^6^. But how the heart alters mechanotransductive signaling against a background of rhythmic contraction and dilatation is obscure.

The intracellular Ca^2+^ concentration plays a key role in receptor-stimulated sustained Nox2 activation, and local Ca^2+^ influx through receptor-operated TRPC channels has been implicated in the initiation and progression of maladaptive cardiac remodeling in rodents. Among the TRPC1-C7 subfamily, TRPC3 and TRPC6 participate in pressure overload-induced myocardial hypertrophy in mice^7^,^8^. In addition, pharmacological inhibition of TRPC3 attenuated oxidative stress and left ventricular (LV) dysfunction in mice with dilated cardiomyopathy^9^. Although TRPC1 and TRPC6, but not TRPC3, reportedly possess mechanosensitive properties^10^, it is circumstantially TRPC3 that participates in mechanical stretch-induced ROS production in neonatal rat cardiomyocytes (NRCMs)^9^. How TRPC3-mediated Ca^2+^ influx specifically encodes signals to activate Nox2-dependent mechanotransduction is unclear.

Recent studies using TRPC3-deficient C57BL/6 J mice have shown that selective inhibition of TRPC3 does not mitigate the LV hypertrophy induced by pressure overload, though deletion of multiple TRPC channels, including TRPC3/C6 and TRPC1/C4, suppresses LV hypertrophy in mice^8^,^11^. From these studies, however, it is not clear whether pressure overload was induced equally in all TRPC-deficient mice.

Here we demonstrate that TRPC3 participates in pressure overload-induced LV dysfunction in 129 Sv mice. Furthermore, TRPC3 acts as a PRROS that stabilizes Nox2 activity through physical interaction, leading to amplification of ROS-dependent maladaptive signaling induced by mechanical stretch during diastolic filling in cardiomyocytes.

## Results

### TRPC3 deletion prevents mechanical stress-induced ROS production and left ventricular dysfunction in pressure-overloaded heart

**Figure 1:**
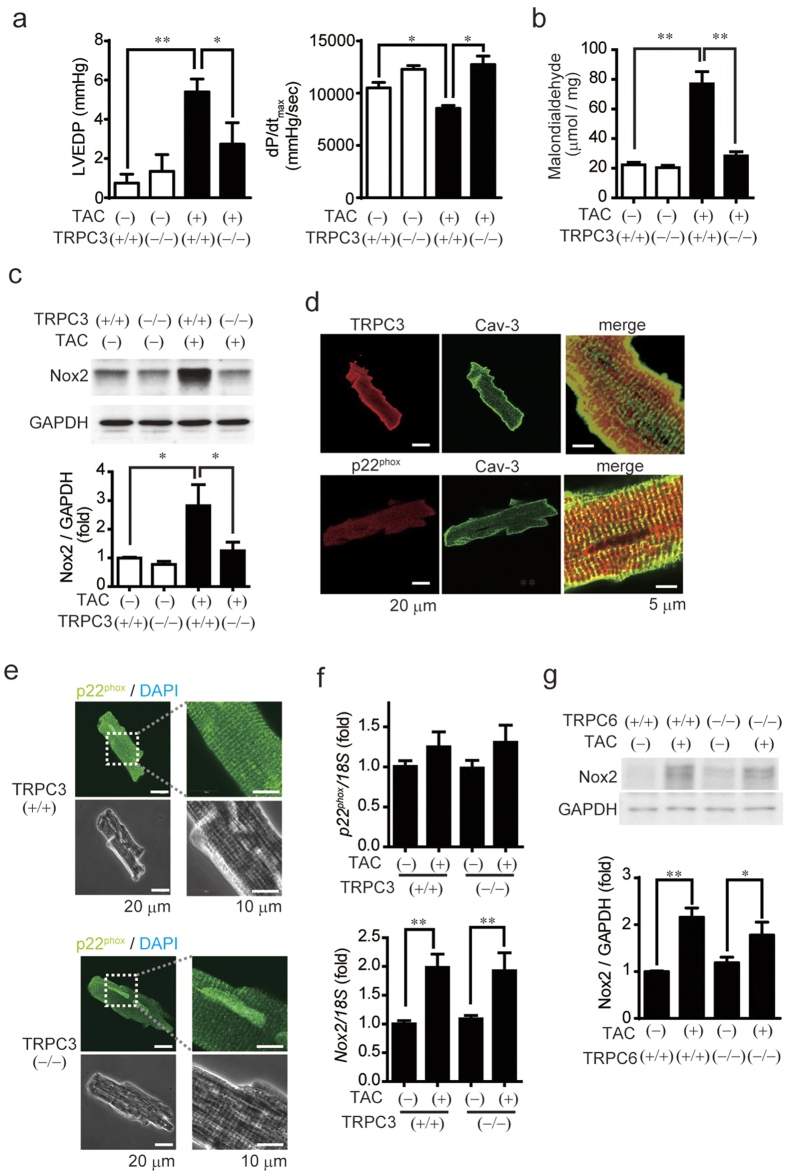
TRPC3 deletion suppresses TAC-induced LV dysfunction and dilation through Nox2 inhibition. (**a**) Left ventricular end-diastolic pressure (LVEDP; left) and dP/dT_max_ (right) in TAC-operated TRPC3^(+/+)^ (n = 13) and TRPC3^(−/−)^ (n = 12) mice 6 week post-operation. (**b**) Myocardial malondialdehyde concentrations 1 week after TAC (n = 4). (**c**) Abundance of Nox2 protein in TRPC3^(+/+)^ and TRPC3^(−/−)^ hearts 1 week after TAC (n = 3). (**d**) Representative immunofluorescence images of TRPC3, p22^phox^, and caveolin-3 (Cav-3) in adult mouse cardiomyocytes isolated from muscle LIM protein-deficient hearts. (**e**) Representative immunofluorescence images of p22^phox^ in adult mouse cardiomyocytes: green, anti-p22^phox^; blue, DAPI. (**f**) Relative abundances of p22^phox^ and Nox2 mRNA in mouse hearts 1 week after TAC (n = 4). (**g**) Abundance of Nox2 protein in TRPC6^(+/+)^ and TRPC6^(−/−)^ hearts 1 week after TAC (n = 3). Error bars, s.e.m. *P < 0.05, **P < 0.01.

Pressure overload induced by transverse aortic constriction (TAC) causes heart failure characterized by LV hypertrophy and associated myocardial stiffness. The increase in LV end-diastolic pressure, an index of LV stiffness, and the decrease in LV contractility induced by TAC were significantly lower in TRPC3 deficient (TRPC3^(−/−)^) mice than in wild type (TRPC3^(+/+)^) mice (Fig. 1a and Table 1), though TAC induced LV hypertrophy in both mice (Table 2). Moreover, TAC-induced production of malondialdehyde, an index of oxidative stress, was nearly abolished in TRPC3^(−/−)^ hearts (Fig. 1b). Mechanical stress reportedly increases Nox2 expression in rodent hearts, and Nox2-derived ROS-induced ROS release from mitochondria is believed to mediate pathological remodeling in response to pressure overload^5^,^12^. TAC significantly increased Nox2 protein in TRPC3^(+/+)^ hearts, but not in TRPC3^(−/−)^ hearts (Fig. 1c). TRPC3 was localized in both cell surface and intracellular membrane, which was merged well with sarcolemmal and transverse-tubule (T-tubule) marker caveolin-3 in isolated adult cardiomyocytes from mice with dilated cardiomyopathy (Fig. 1d) 9. p22^phox^, an essential partner of Nox2 to form stable heteromeric complex, was localized exclusively in T-tubule. These results indicate that both TRPC3 and p22^phox^ proteins are co-localized in T-tubule in cardiomyocytes. The abundances of Nox2 and its partner p22^phox^ in normal TRPC3^(−/−)^ hearts were slightly lower than in normal TRPC3^(+/+)^ hearts (Fig. 1c,e). By contrast, detected amounts of Nox2 and p22^phox^ mRNA did not differ between TAC-operated TRPC3^(−/−)^ and TRPC3^(+/+)^ hearts (Fig. 1f), indicating that TRPC3 contributes to stabilize Nox2 and p22^phox^ protein in mouse hearts. TRPC6, a close homologue of TRPC3, has been also reported to play a role in cardiac remodeling^13^. However, the abundance of Nox2 protein in TAC-operated TRPC6^(−/−)^ mouse hearts was increased to the same extent as that in TAC-operated TRPC6^(+/+)^ mouse hearts (Fig. 1g). These data suggest that TRPC3 specifically contributed to the upregulation of Nox2 in pressure-overloaded heart.

**Figure 2:**
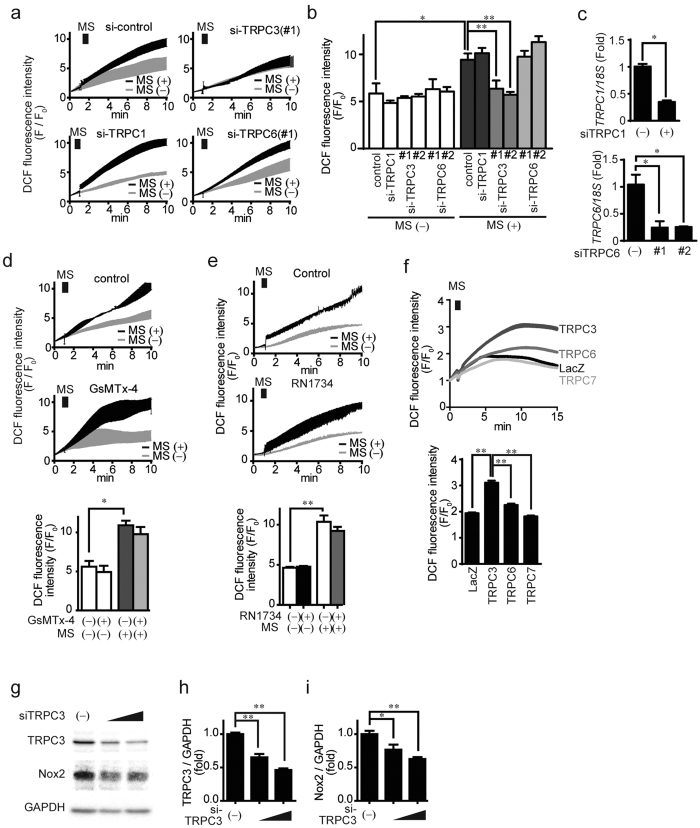
TRPC3 plays a critical role in Mechanical stretch-induced ROS production. (**a**,**b**) Effects of siRNA targeting TRPC1, C3 or C6 on mechanical stretch (MS)-induced ROS production (n = 3). (**c**) mRNA expression of either TRPC1 or TRPC6 in NRCM transfected with siRNAs against either TRPC1 or TRPC6, respectively (n = 3). (**d,e**) Time courses of MS-induced ROS production in NRCMs treated with GsMTx-4 (1 μM; (**d**) or TRPV4 inhibitor (RN1734, 50 μM; (**e**) Reagents were added to cells 5 min before MS (n = 3). (**f**) MS-induced ROS production in TRPC(1–7)-deficient MEF cells expressing TRPC3, TRPC6, TRPC7, or LacZ (n = 30). Data are representative of three independent experiments. (**g–i**) Effect of TRPC3 siRNA on the protein abundances of TRPC3 (**h**) and Nox2 (**i**) protein expressions in NRCMs (n = 3). Error bars, s.e.m. *P < 0.05, **P < 0.01.

To investigate the functional relationship between TRPC3 and ROS production, NRCMs were subjected to mechanical stretch to mimic the stretching of the LV myocardium induced by pressure overload. Mechanical stretch-induced ROS production in NRCMs was nearly completely suppressed by TRPC3 knockdown, but not by knockdown or pharmacological inhibition of other mechanosensitive TRP channels (TRPC1, TRPC6^10^ and TRPV4^14^) (Fig. 2a–e). Additionally, mechanical stretch elicited no apparent ROS production in TRPC (1–7)-deficient mouse embryonic fibroblasts (MEFs), though adding TRPC3 back to TRPC(1–7)-KO MEFs markedly increased ROS production to a level similar to that in wild type MEFs (Fig. 2f). This indicates that among mechanosensitive and mechano-activated TRP channels, specifically TRPC3 mediates mechanical stress-induced ROS production. In addition, TRPC3 knockdown significantly reduced endogenous Nox2 in NRCMs (Fig. 2g–i), suggesting TRPC3 deletion may suppress mechanical stress-induced ROS production in rodent hearts in part by destabilizing Nox2.

### TRPC3 positively regulates the expression of Nox2

**Figure 3:**
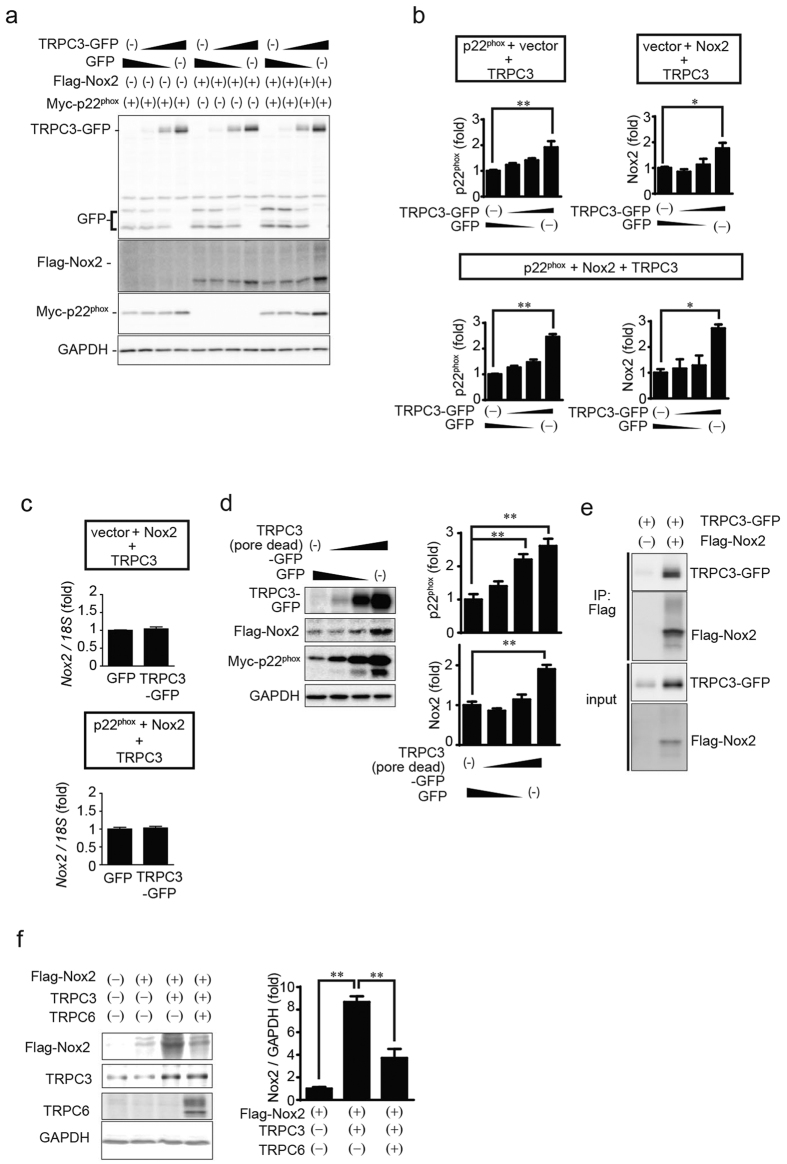
TRPC3 forms a stable ternary complex with Nox2 and p22^phox^. (**a**,**b**) Expression of Nox2 and p22^phox^ proteins in HEK293 cells that express a different combination of TRPC3-GFP and GFP. Results of a quantitative analysis are shown in (**b**) (n = 3). (**c**) Nox2 mRNA amounts in HEK293 cells co-expressing Nox2 with GFP or TRPC3-GFP (n = 3). (**d**) Increased Nox2 and p22^phox^ protein in HEK293 cells co-expressing pore-dead mutant of TRPC3 (n = 3). (**e**) Interaction of TRPC3 with Nox2 in HEK293 cells. Immunoprecipitation was performed using an anti-flag antibody. (**f**) Nox2 protein expression in HEK293 cells expressing TRPC3 alone or co-expressing TRPC3 and TRPC6 (n = 3). Error bars, s.e.m. *P < 0.05, **P < 0.01.

**Figure 4:**
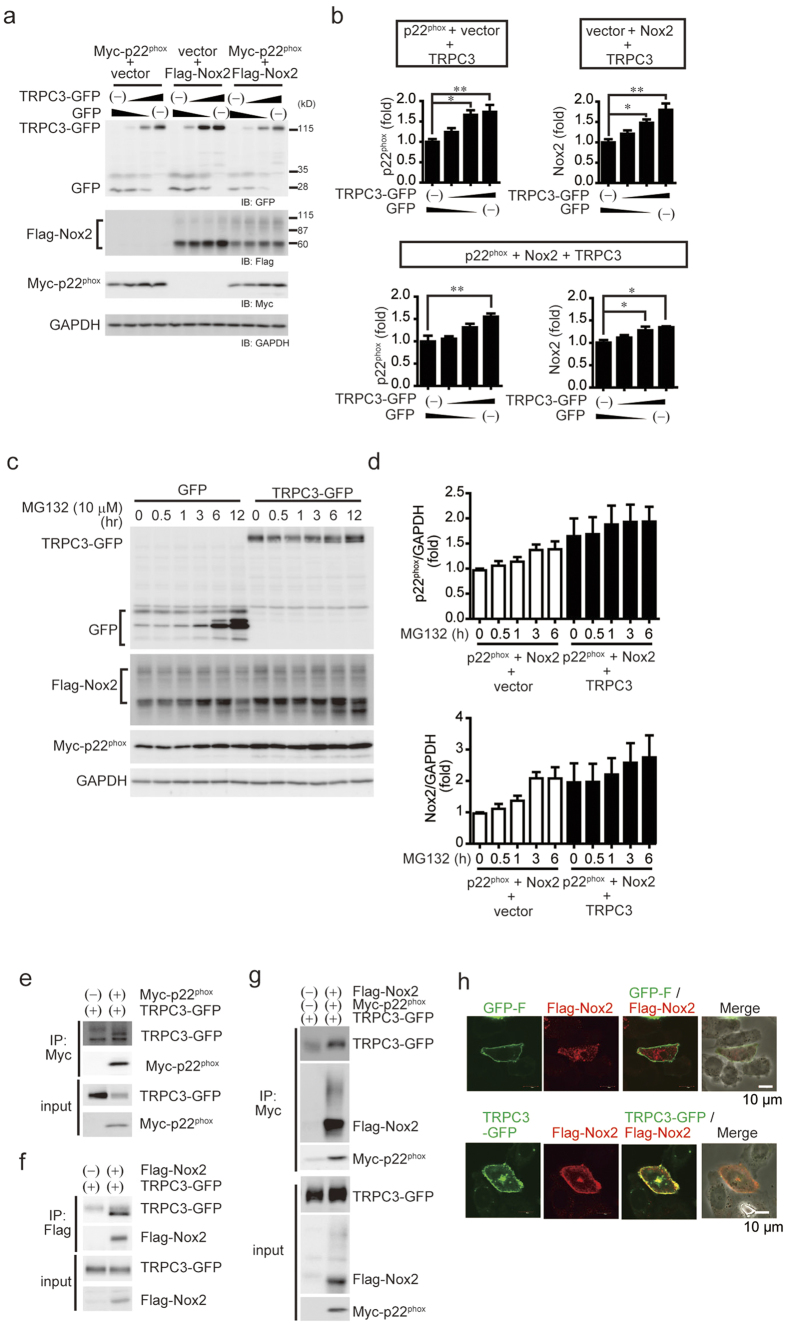
TRPC3 forms a stable ternary complex with Nox2 and p22^phox^ proteins in endogenously p22^phox^ -absent CHO cells. (**a**) Expression of Nox2 and p22^phox^ proteins in CHO cells that express a different combination of TRPC3-GFP and GFP. (**b**) Results of quantitative analysis (n = 3). (**c**) Expression of Nox2 and p22^phox^ co-expressed with either GFP or TRPC3-GFP in MG132 (10 μM)-treated CHO cells. (**d**) Graphs depict the relative expression of either Nox2 or p22^phox^ protein to that in non-treated cells. Band intensities were normalized by GAPDH. (**e–g**) Interaction of TRPC3 with p22^phox^ and Nox2 in CHO cells. (**h**) Localization of Nox2 in CHO cells co-expressing Nox2 with TRPC3-GFP (or GFP-F). Error bars, s.e.m. *P < 0.05, **P < 0.01.

**Figure 5:**
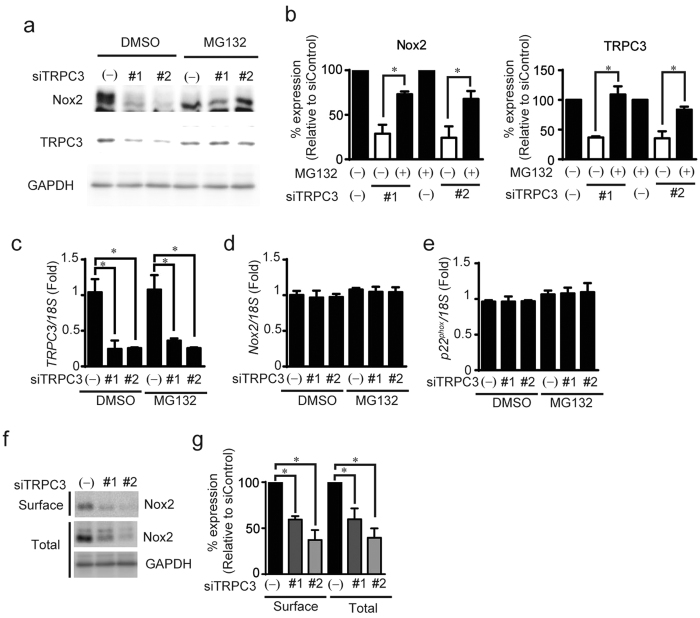
TRPC3 prevents Nox2 protein from proteasomal degradation. (**a–e**) Abundances of Nox2 protein (**a**,**b**) and mRNAs of TRPC3 (**c**), Nox2 (**d**), and p22^phox^ (**e**) in NRCM transfected with siRNAs targeting TRPC3 with or without MG132. Cells were treated with siRNAs and MG132 (1 μM) simultaneously (n = 3). (**f**,**g**) Effect of siRNA targeting TRPC3 on Nox2 protein abundance in cell surface (Surface) and total lysates (Total) from NRCMs (n = 3). GAPDH was used as an internal control. Error bars, s.e.m. *P < 0.05.

Interaction of p22^phox^ with Nox is required for Nox protein maturation, stabilization and subcellular localization^15^,^16^. We therefore used HEK293 cells and CHO cells, which lack endogenous p22^phox^, to test whether TRPC3 stabilizes Nox2 and p22^phox^ by forming a ternary complex. Expression of EGFP-fused TRPC3 (TRPC3-GFP), but not GFP, significantly increased the abundances of Nox2 and p22^phox^ protein (Fig. 3a,b), without increasing amount of Nox2 mRNA (Fig. 3c). Pore-dead mutant of TRPC3 also stabilized Nox2 protein as much as wild type TRPC3, suggesting that TRPC3 stabilizes Nox2 independently of TRPC3 channel activity (Fig. 3d). This TRPC3-dependent Nox2 stabilization was also observed in p22^phox^-deficient CHO cells (Fig. 4a,b), suggesting that TRPC3 stabilizes Nox2 independently of the Nox2-p22^phox^ interaction. Co-expression of TRPC6 with TRPC3 canceled the increase in Nox2 protein abundance evoked by TRPC3 single expression (Fig. 3f). This result supports the fact that TAC-induced Nox2 upregulation is not attenuated in TRPC6^(−/−)^ hearts, and indicates the specific role of TRPC3 as PRROS. The proteasome inhibitor MG132 increased Nox2 and p22^phox^ in TRPC3-negative cells nearly to the levels seen in TRPC3-expressing cells (Fig. 4c,d), which suggests both Nox2 and p22^phox^ are continuously degraded via a proteasome-dependent pathway. Immunoprecipitation analysis revealed that TRPC3-GFP associates with flag-tagged Nox2 (Flag-Nox2) in HEK293 cells (Fig. 3d), and with Flag-Nox2 and myc-tagged p22^phox^ (myc-p22^phox^) in CHO cells (Fig. 4e–g). Co-expression of TRPC3-GFP led to increase in plasma membrane surface expression of Flag-Nox2 (Fig. 4h). Proteasomal degradation of endogenous Nox2 was also observed in TRPC3-silenced NRCMs, and the detected amounts of TRPC3 and Nox2 proteins, but not mRNAs, were restored by MG132 treatment, despite significant reductions in TRPC3 mRNA (Fig. 5a–e). In addition, the decreasing rate of detected amount of Nox2 in surface membrane fraction was equivalent to that in total NRCM lysate (Fig. 5f,g), suggesting that TRPC3 does not promote the translocation of Nox2 to plasma membrane. As p22^phox^ predominantly regulates the subcellular localization of Nox enzymes^15^, these data suggest that TRPC3 protects Nox2 from proteasome-dependent degradation by unknown mechanism different from p22^phox^-dependent Nox2 modification.

### Reciprocal and functional coupling between TRPC3 and Nox2

**Figure 6:**
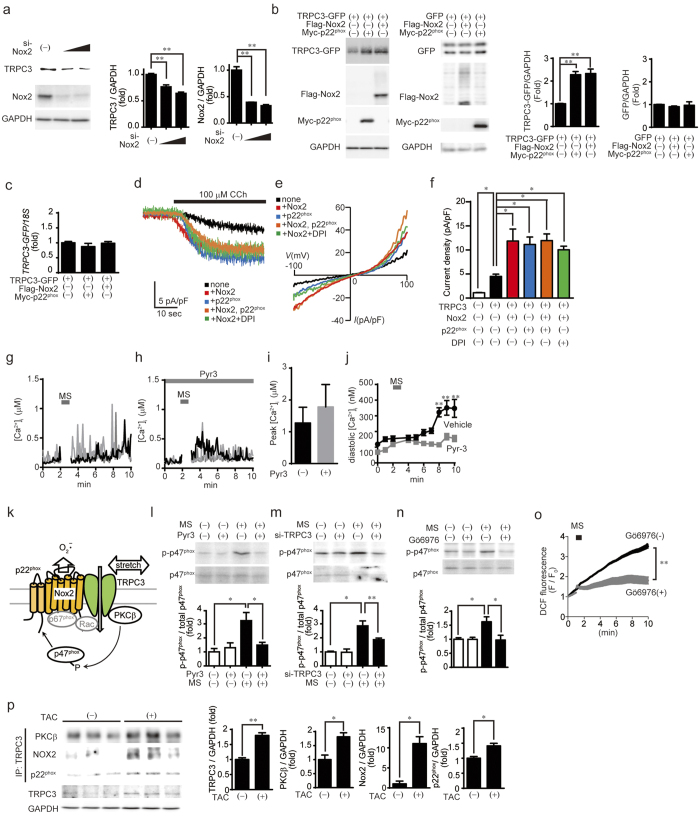
Formation of a TRPC3/Nox2 complex promotes TRPC3 channel activity through stabilization at the plasma membrane. (**a**) Effect of Nox2 siRNA on expression of TRPC3 in NRCMs (n = 3). (**b**) Representative images showing the levels of TRPC3-GFP and GFP expression in HEK293 cells co-expressing p22^phox^ or Nox2 (n = 3). (**c**) Expression of TRPC3-GFP mRNA in HEK293 cells co-expressing p22^phox^ or Nox2 (n = 3). (**d–f**) Representative time courses of TRPC3 currents (**d**) and the current-voltage (I-V) relationships (**e**) and peak TRPC3 current densities at −60 mV (**f**) induced by 100 μM carbachol (CCh) in HEK293 cells expressing TRPC3-mCherry alone or with p22^phox^, Nox2, both p22^phox^ and Nox2, or Nox2 treated with DPI. DPI (0.3 μM) was treated 1 min before CCh stimulation. (**g**,**h**) Representative Ca^2+^ responses in the presence (**g**) or absence (**h**) of pyrazole-3 (Pyr3, 1 μM) upon mechanical stretch (MS) application. (**i**) Peak Ca^2+^ increases after MS in NRCMs treated with (n = 61) or without Pyr3 (n = 78). (**j**) Changes of minimal [Ca^2+^]_i_ before and after MS application. Minimal [Ca^2+^]_i_ from Ca^2+^ responses in every 1 min were analyzed and represented as diastolic [Ca^2+^]_i_. (**k**) Schematic images showing phosphorylation of p47^phox^ via TRPC3-PKCβ activation induced by MS in the heart. (**l–n**) Effects of TRPC3 (**l**,**m**) or PKCβ (**n)**; 10 μM Gö6976) inhibitors on p47^phox^ phosphorylation induced by MS in NRCMs (n = 3). (**o**) MS-induced ROS generation in NRCMs treated with a PKCβ inhibitor (n = 3). (**p**) Co-immunoprecipitation of TRPC3 with PKCβ, Nox2 and p22^phox^ in mouse hearts 1week after TAC operation (n = 3). Error bars, s.e.m.*P < 0.05, **P < 0.01.

Interestingly, Nox2 knockdown also reduced detected TRPC3 in NRCMs (Fig. 6a). We therefore examined whether TRPC3 stability and thus channel activity is also enhanced by co-expression of p22^phox^ and/or Nox2. The abundance of TRPC3-GFP, but not GFP, was significantly increased by co-expression of either myc-p22^phox^ or Flag-Nox2 in HEK293 cells (Fig. 6b), whereas TRPC3-GFP mRNA was equally increased in HEK293 cells, with or without myc-p22^phox^ and/or Flag-Nox2 (Fig. 6c). Co-expression of TRPC3 with p22^phox^ and/or Nox2 also significantly enhanced the inward current induced by muscarinic receptor stimulation (Fig. 6d–f). This enhanced TRPC3 current was insensitive to diphenyleneiodonium (DPI), a pharmacological inhibitor of Nox2 (Fig. 6d–f). These results suggest that formation of a TRPC3/p22^phox^/Nox2 complex enhances TRPC3 channel activity by increasing mature TRPC3 proteins on the plasma membrane. Importantly, pharmacological inhibition of TRPC3 using pyrazole-3 had no impact on the increases in global Ca^2+^ concentrations induced by mechanical stretch, but suppressed an increase in basal Ca^2+^ concentration (Fig. 6g–j), suggesting that TRPC3 contributes to mechanical stress-induced background Ca^2+^ entry in NRCMs.

We previously reported that TRPC3 interacts with phospholipase Cγ2 and protein kinase C (PKC) β in B lymphocytes and regulates their activities by mediating Ca^2+^ influx^17^,^18^. PKCβ is known to activate Nox2 via phosphorylation of the Nox2-activating p47^phox^ subunit^19^ (Fig. 6k). Mechanical stretch of NRCMs increased p47^phox^ phosphorylation, and both p47^phox^ phosphorylation and mechanical stretch-induced ROS production were significantly suppressed by inhibition of TRPC3 and PKCβ (Fig. 6l–o). This suggests the involvement of PKCβ-dependent p47^phox^ phosphorylation in TRPC3-induced Nox2 activation. Indeed, endogenous TRPC3 complexed with PKCβ as well as Nox2 and p22^phox^, and the abundance of these proteins was increased in TAC-operated hearts (Fig. 6p). These results indicate a close interdependence between TRPC3 and the Nox2/p22^phox^ complex that affects not only protein stability but also cation influx and ROS production (Fig. 6k).

### Stabilization of Nox2 by physical interaction with TRPC3 at its C-terminus

**Figure 7:**
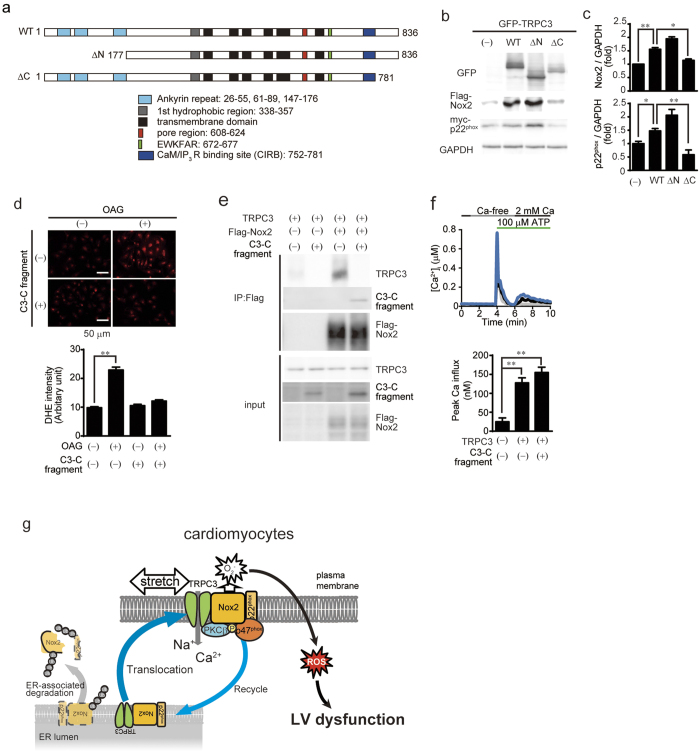
Physical interaction between TRPC3 and Nox2 is critical for stabilization of Nox2. (**a**) Schematic illustration of TRPC3 terminal deletion mutants. (**b**,**c**) Expression of Nox2 and p22^phox^ co-expressed with TRPC3 deletion mutants in HEK293 cells (n = 3). (**d**) OAG-induced ROS production in NRCMs expressing Nox2-interacting TRPC3 C-terminal fragment (C3-C fragment) (n = 20–28). (**e**) Co-immunoprecipitation of TRPC3 with Nox2 in the presence or absence of C3-C fragment. Representative blot from three independent experiments was shown. (**f**) ATP (100 μM)-induced Ca^2+^ responses in HEK293 cells expressing TRPC3 with or without C3-C fragment (n = 35–51). Timing of solution exchanges were indicated by horizontal bars above the graph. (**g**) Model of the regulation of TRPC3-Nox2 stability and induction of LV dysfunction induced by diastolic stretch of cardiomyocytes. Error bars, s.e.m.*P < 0.05, **P < 0.01.

The cytosolic domains of TRP channels are reportedly important for formation of protein signaling complexes^18^,^20^. We found that interaction of TRPC3 with Nox2 was significantly reduced by deletion of a TRPC3-specific C-terminal sequence (781–836 aa), but not by deletion of the N-terminal region (Fig. 7a–c). Thus, a TRPC3-specific C-terminal region may be essential for the functional interaction with Nox2. To demonstrate the importance of physical interaction between TRPC3 and Nox2, GFP-fused Nox2-interacting C-terminal fragment of TRPC3 (C3-C fragment) was constructed. Stimulation of TRPC3 with 1-oleoyl-2-acetyl-sn-glycerol (OAG)^9^ significantly increased ROS production in GFP-expressing NRCMs, while this ROS production was almost completely suppressed in C3-C fragment-expressing NRCMs (Fig. 7d). We confirmed that C3-C fragment could interfere with the association of Nox2 to TRPC3 without reducing TRPC3 channel activity in HEK293 cells (Fig. 7e,f). These results suggest that TRPC3-Nox2 interaction *per se* is critical for stabilization of Nox2 protein complex, and activation of TRPC3 is important for Nox2-dependent ROS production induced by mechanical stretch (Fig. 7g).

## Discussion

The physiological significance of Nox-derived ROS production is well established in host defense of macrophages, where Nox activities are precisely controlled by the enzyme’s expression level and negative regulatory mechanisms, such as endosomal degradation via interaction with negative regulator of ROS (NRROS)^21^. This is essential to maintain redox homeostasis in macrophages, which strongly express Nox2. Because basal Nox expression is much lower in cardiomyocytes than macrophages, positive regulatory mechanisms affecting Nox2 are important for adapting the heart to mechanical stress. We demonstrated that TRPC3, originally identified as a major component of receptor-operated cation channels, specifically increases Nox2 activity during mechanical stretch in cardiomyocytes. Moreover, the interaction of Nox2 with TRPC3 protects Nox2 from proteasome-dependent degradation, making TRPC3 a positive regulator of ROS, or PRROS. Considering that NRROS contribute to degradation-dependent “tuning” of Nox2 for regulating ROS production in phagocytes, amplification of Nox2-mediated ROS production by PRROS may also be physiologically important for increasing cardiac contractility in response to hemodynamic overload^4^.

We have identified how TRPC3-mediated local Ca^2+^ influx specifically encodes signals to induce maladaptive cardiac remodeling. TRPC3 contributes to the transition from adaptive hypertrophy to maladaptive hypertrophy induced by pressure overload in mice. As the deletion of Nox2 gene reportedly attenuates pressure overload-induced cardiac dysfunction through suppressing fibrosis, but not hypertrophy, in TAC-operated mice^12^, TRPC3-Nox2 communication can explain the mechanism how TRPC3-mediated local Ca^2+^ influx specifically encodes maladaptive hypertrophic signaling in mice.

TRPC3-deficient myocardium apparently has a higher susceptibility to TAC than wild-type myocardium, since TAC-induced increases in LV systolic pressure was significantly higher in TRPC3-deficient hearts than wild-type hearts (Table 1). This implies that TRPC3 sets limits on the hypertrophic growth of the myocardium evoked by pressure overload *in vivo*. However, this idea contradicts our previous finding that TRPC3 participates in the development of myocardial hypertrophy *in vivo*^7^,^9^ and *in vitro*^22^. Chronic increases in the diastolic Ca^2+^ concentration exacerbates heart failure as well as LV remodeling^23^, and TRPC3-mediated background Ca^2+^ entry contributes to the mechanical stretch-induced increase in the basal (i.e., diastolic) Ca^2+^ concentration in NRCMs. Thus, TRPC3-mediated ROS signaling via functional coupling to Nox2 may be critical for limiting hypertrophic overgrowth of cardiomyocytes and for inducing fibrogenic signaling to stiffen the heart to compensate for mechanical overload.

The functional communication between several TRP channels and ROS has been reported previously, though all of those studies focused on the Nox-TRP axis, including TRPC6 and TRPM2 in tissues strongly expressing Nox^24^,^25^. The positive regulation of Nox2 by TRPC3 observed in the present study suggests that there is indirect crosstalk between TRPC3 and other ROS-activated TRP channels through Nox2 activation. In phagocytes, which strongly express Nox2, sustained Ca^2+^ entry through Orai1 store-operated Ca^2+^ channels^26^ is sufficient to increase Nox2 activity in response to bacterial infection. Voltage-dependent H^+^ channels^27^ also contribute to ROS production by acting to balance the charge on the membrane. However, this simple machinery is not conserved in cardiac cells, where Nox2 expression is comparatively weak and the physiological function of ROS is very different from that in phagocytes. Formation of a functional protein complex between Nox2 and TRPC3 is thus necessary for specific regulation of Nox2-dependent ROS signaling in the context of cardiac mechanotransduction. Several reports have suggested that TRPC3 expression level is transcriptionally upregulated at the onset of cardiac remodeling^28^,^29^. Mechanical stretch caused by LV chamber dilation increases TRPC3-mediated Ca^2+^ influx activity (Fig. 6g–j). Our finding suggests that increase in TRPC3 channel activity induced by mechanical stress may initiate TRPC3 upregulation, which leads to amplification of Nox2-dependent ROS production through formation of TRPC3-Nox2 stable complex, resulting in ROS-dependent LV dysfunction, but further studies must be required to elucidate the mechanism underlying TRPC3 upregulation at the onset of cardiac remodeling.

Chronic heart failure, emerging as loss of LV function following maladaptive tissue remodeling, is a leading cause of death worldwide. Pharmacological inhibition^7^ or genetic ablation of TRPC3 is found to prevent mechanical stress-induced maladaptive cardiac remodeling through proteasomal degradation of Nox2 in cardiomyocytes (Fig. 7g). Nox2-derived ROS is reported to mediate a burstic Ca^2+^ spark induced by physiological diastolic stretch of low TRPC3-expressing adult cardiomyocytes^4^, while Nox2 upregulation aggravates cardiac hypertrophy and fibrosis^30^. As we could not have found any functional abnormality in normal TRPC3^(−/−)^ hearts so far, the TRPC3-Nox2 coupling might be restricted in the failing heart. Because both TRPC3 and Nox2 are abundantly expressed in immune cells and play important roles in innate immunity^17^,^18^,^27^, targeting inhibition of TRPC3 channel activity or Nox2 enzymatic activity may cause adverse immunosuppressive effect. Therefore, tuning of TRPC3-Nox2 stability by disrupting TRPC3-Nox2 interaction will be a new therapeutic strategy to improve chronic heart failure.

## Methods

### Animals

All protocols using mice and rats were reviewed and approved by the ethics committees at the National Institutes of Natural Sciences or the Animal Care and Use Committee, Kyushu University, and were performed according to the institutional guidelines concerning the care and handling of experimental animals. 129 Sv mice with homozygous deletion of the gene encoding TRPC3 were provided by the Comparative Medicine Branch, National Institute of Environmental Health Sciences, Research Triangle Park, North Carolina 27709. Genotyping was performed using PCR primers; TRPC3-A 5′-GAATCCACCTGCTTACAACCATGTG-3′ and TRPC3-B 5′-GGTGGAGGTAACACACAGCTAAGCC-3′. The PCR was performed using Phusion High-Fidelity DNA polymerase (Thermo Scientific). Mice were maintained in specific-pathogen-free area under a 12 h/12 h light/dark cycle. C57BL/6 J mice were purchased from SLC. Sprague-Dawley rats were purchased from Kyudo or SLC.

### Pressure overload study in mice

Pressure overload was induced as described previously^9^,^31^. Male mice, 6–8 weeks old, were used for these experiments. Cardiac pressure overload was induced by TAC. Briefly, mice were anaesthetized using a mixture of domitor (Zenoaq), midazolam (Sando) and butorphanol (Meiji Seika Pharma). After orotracheal intubation and ventilation, an intercostal space was opened. The transverse aorta was then exposed and constricted between the brachiocephalic artery and left carotid artery to the width of a 27-G needle using a 5-0 silk braid. Sham treatment was performed similarly but without constriction of the silk braid. An osmotic minipump (model 2004 (Alzet)) filled with vehicle (polyethylene glycol) or pyrazole-3 (3 and 10 μg kg^−1^ day^−1^) was implanted intraperitoneally 3 days after the TAC procedure^31^. To assess expression of hypertrophy- and fibrosis-related genes, an osmotic minipump was implanted intraperitoneally 1 week before TAC^9^. Six weeks after TAC, LV hemodynamic parameters were assessed in anesthetized mice using a micronanometer catheter (Millar 1.4 F, SPR 671, Millar Instruments).

### Cell culture

HEK293 cells were cultured in DMEM supplemented with 10% FBS and 1% penicillin and streptomycin. TRPC(1–7)-deficient MEFs were cultured in DMEM supplemented with 10% FBS, 2 mM L-glutamine, and 1% penicillin and streptomycin. The MEF cells were infected with Adenovirus encoding LacZ, TRPC3, TRPC6 or TRPC7 in serum-free DMEM as described previously^22^ and plated onto fibronectin-coated stretch chambers. Primary human cardiac fibroblasts were purchased from Lonza and cultured according to manufacturer’s instruction.

### Plasmid DNA and transfection

Detailed information of TRPC3-GFP^18^, pore-dead mutant of TRPC3-GFP^18^, Flag-Nox2^32^, and myc-p22^phox ^33 were described elsewhere. C-terminal deletion mutants of TRPC3 and Nox2-interacting fragment of TRPC3 C-terminus were PCR amplified and cloned into pEGFP-N1 vector (Clontech). Plasmid DNAs were transfected to HEK293 and CHO cells by X-tremeGENE 9 transfection reagent (Roche) and to NRCMs by Lipofectamine 2000 (Invitrogen) according to manufacturer’s instruction.

### Isolation of cardiomyocytes from neonatal rats

Rat pups were sacrificed on postnatal day 1–3, after which the left ventricles were removed and minced. The minced tissue was pre-digested in 0.05% trypsin-EDTA (Gibco) over night at 4 °C and then digested in 1 mg ml^−1^ collagenase type 2 (Worthington) in PBS for 30 min at 37 °C. The dissociated cells were plated in a 10-cm culture dish and incubated at 37 °C in a humidified atmosphere (5% CO_2_, 95% air) for 1 h in DMEM containing 10% FBS and 1% penicillin and streptomycin. Attached cells were cardiac fibroblast and cultured in same medium. Floating cells were collected and plated into gelatin-coated culture dishes or laminin-coated stretch chamber dishes at a density of around 1.5 × 10^5^ cells/cm^2^. After 24 h, the culture medium was changed to serum-free DMEM. For protein knockdown, cells were transfected with siRNAs (100 nM) using Lipofectamine 2000 for 72 h. Information on the sequences used is provided in Supplementary Table 1.

### Measuring mRNA expression in cells and tissues

Total RNA was isolated from frozen mouse heart samples using a RNeasy Fibrous Tissue Mini Kit (Qiagen) or from cardiomyocytes using a RNeasy Mini Kit (Qiagen) according to the manufacturer’s instructions. Quantitative real-time PCR was performed using an ABI PRISM 7500 Real-Time PCR System (Applied Biosystems) and a OneStep RT-PCR Kit (Qiagen) or Power SYBR green PCR master mix (Applied Biosystems) according to the manufacturer’s instructions. The primers are described in Supplemental Table 2.

### Measurement of intracellular Ca increase and ROS production

Measurement of intracellular Ca increases were performed with Fura-2/AM (Dojin) as previously described^18^. Intracellular ROS concentrations were measured using 2′,7′-dichlorodihydrofluorescein diacetate (H_2_DCFDA) (Molecular Probes), as described previously with slight modification^9^,^22^. H_2_DCFDA was loaded into cardiomyocytes on stretch chamber dishes using the following procedure. After aspirating the culture medium from the dishes and washing the cells with DMEM, freshly prepared 5 μM H_2_DCFDA or Fura-2AM diluted in DMEM was added to the dishes and incubated for 30 min at 37 °C in a humidified atmosphere (5% CO_2_, 95% air). The dye solution was then replaced with HEPES-buffered saline solution (HBSS) containing 118 mM NaCl, 5.4 mM KCl, 10 mM glucose, 10 mM HEPES (pH 7.4), 1.2 mM MgSO_4_ and 2 mM CaCl_2_. The stretch chamber dish was mounted in a Stretch System for Microscopes STB-150 (Strex) on the stage of an upright fluorescence microscope (Olympus). The mechanical stretch entailed application of a 20% stretch ratio for 3 s at a stretching speed of 80 mm/sec. Fluorescence images were recorded and analyzed using a video image analysis system (Aquacosmos, Hamamatsu Photonics). DHE stating in NRCMs was performed as previously described^9^.

### Measurement of malondialdehyde

Total cardiac malondialdehyde (MDA) was assessed in mice as described previously using a Bioxytech MDA-586 kit (OxisResearch) according to the manufacturer’s instructions^5^. Briefly, frozen mouse heart samples were weighed and homogenized in potassium phosphate extraction (KPE) buffer (pH 7.5) containing 100 mM potassium phosphate, 5 mM EDTA, 0.1% (v/v) Triton X-100 and 0.6% sulfosalicylic acid and 5 mM BHT, and the lysates were clarified by centrifugation at 15,000 r.p.m. for 10 min at 4 °C. Samples of supernatant were then allowed to react with N-methyl-2-phenylindole (NMPI) at an acidic pH at 45 °C for 1 h, after which the samples were clarified by centrifugation at 15,000 r.p.m. for 10 min at 4 °C and read at 586 nm using a Spectra Max i3 (Molecular Devices). MDA concentrations were estimated using a standard curve derived using 0.5 to 4.0 μM standard MDA.

### Immunoprecipitation and western blotting

HEK293 cells were lysed in IP lysis buffer containing 20 mM Tris-HCl (pH 7.5), 1% (v/v) Triton X-100, 150 mM NaCl, 10 mM Na_2_HPO_4_, 0.2 mM Na_3_VO_4_, 10 mM NaF, and a protease inhibitor cocktail. Cell lysates were sonicated and clarified by centrifugation at 13,000 r.p.m. for 10 min at 4 °C, and the supernatants were equalized for total volume (1 ml) and the amount of protein (1 mg). Myc-tagged proteins were immunoprecipitated from supernatants using anti-myc tag antibody (1.5 μg) in the presence of a 20-μl bed volume of protein A Sepharose beads (GE Healthcare). Flag-tagged proteins were immunoprecipitated from supernatants containing a 20-μl bed volume of anti-flag M2 Affinity Gel (Sigma Aldrich). myc-tagged proteins were immunoprecipitated from supernatants using anti-myc antibody (Merck) in the presence of a 20-μl bed volume of protein G Sepharose beads (GE Healthcare). The immune complexes were washed three times with lysis buffer, suspended with SDS sample buffer containing 0.1 M DTT, and incubated at room temperature for 1 h. To measure levels of p22^phox^ and Nox2 expression, cells plated in 12-well dishes transiently transfected with Flag-tagged Nox2 and myc-tagged p22^phox^ (10:10 ratio) with a gradient TRPC3-GFP/GFP ratio (0:10, 1:9, 5:5, 10:0) using X-tremeGENE 9 DNA Transfection Reagent according to the manufacturer’s instructions. The total amount of DNA was adjusted to 0.75 μg with empty vector. Forty-eight hours after transfection, cells were harvested in lysis buffer containing 50 mM Tris-HCl (pH 7.5), 5 mM EDTA, 1% (v/v) Triton X-100, 0.2 mM Na_3_VO_4_, 10 mM NaF, and a protease inhibitor cocktail. To analyze expression of endogenous TRPC3 and Nox2 in NRCMs, total membrane fraction was isolated as described previously^18^. Surface protein biotinylation with cell surface protein isolation kit (Pierce) was conducted according to manufacturer’s instruction. Mouse hearts were homogenized in lysis buffer, after which the cell lysates were sonicated and clarified by centrifugation at 13,000 r.p.m. for 10 min at 4 °C. The supernatants were equalized for total volume (1 ml) and amount of protein (1 mg), suspended in SDS sample buffer containing 0.1 M DTT and incubated at room temperature for 1 h. For western blotting, the samples (15 μg) were fractionated by SDS-PAGE and transferred onto PVDF membrane (Millipore), after which the objective proteins were detected using the indicated antibodies. After incubation with the secondary antibody, the bands were visualized using Western Lightning Plus ECL (PerkinElmer). The images were captured using ImageQuant LAS 4000 (GE healthcare Life Science) and quantification was done using ImageQuant TCL software (GE healthcare Life Science). The following primary antibodies were used: GAPDH (sc-25778), gp91^phox^ (sc-130543), p47^phox^ (sc-17845) and p22^phox^ (sc-20781) from Santa Cruz Biotechnology, flag M2-HRP (A8592) from Sigma Aldrich, GFP (CHIP grade, ab290) from Abcam, myc-tag (05–742) from Merck, phospho p47^phox^ (p-Ser370) (A1171) from Assay Bio Tech, and TRPC3 (ACC-016) from Alomone Labs. A TRPC3 antibody for western blotting was developed by immunizing a rabbit with TRPC3 C-terminal peptide corresponding to amino acids 713–736 of mouse TRPC3. The following secondary antibodies were used: goat anti-rabbit IgG-HRP (sc-2004) and goat anti-mouse IgG-HRP (sc-2005) from Santa Cruz Biotechnology.

### Immunohistochemical staining

For immunohistochemical staining of TRPC3, p22^phox^ and Cav-3 in adult mouse cardiomyocytes, adult mouse ventricular myocytes were isolated from 129 Sv TRPC3^(+/+)^, TRPC3^(−/−)^, or 12-week old muscle LIM protein-deficient mice as previously reported^9^ and pelleted by centrifugation at 500 r.p.m. for 5 min at room temperature. The cell pellets were suspended and fixed in 1% paraformaldehyde in PBS and then washed twice in PBS. The fixed cardiomyocytes were permeabilized using 0.1% Triton X-100 in PBS for 5 min on ice then blocked using 1% FBS in PBS for 1 h at room temperature. Antibodies against TRPC3 (Alomone lab) p22^phox^ (Santa Cruz Biotechnology) and Cav-3 (BD transduction laboratories) were diluted in incubation solution containing 0.01% (v/v) Triton X-100, 5% (w/v) BSA and 3% (v/v) FBS in PBS over night at 4 °C. After incubation with the secondary antibody and nuclear staining using DAPI, images were captured using a confocal laser-scanning microscope (FV-10i, Olympus).

### Whole cell patch clamp

TRPC3 currents were measured using the whole-cell patch-clamp technique with an EPC-10 patch-clamp amplifier (Heka Elektronik)^18^. Patch electrodes with a resistance of 3–4 MΩ (when filled with internal solution) were made from 1.5-mm borosilicate glass capillaries (Sutter Instrument). Voltage-clamp experiments were performed at a holding potential of −60 mV, and recordings were sampled at 2.0 kHz and filtered at 2.9 kHz. To analyze *I-V* relationships, ramp pulses from −100 to 100 mV over 250 ms were applied every 30 s. Cells were allowed to settle in the perfusion chamber in the external solution containing 140 mM NaCl, 5.6 mM KCl, 1 mM MgCl_2_, 2 mM CaCl_2_, 10 mM HEPES and 10 mM glucose (pH 7.4). The pipette solution contained 120 mM CsOH, 120 mM aspartate, 20 mM CsCl, 2 mM MgCl_2_, 5 mM EGTA, 1.5 mM CaCl_2_, 10 mM HEPES, 2 mM ATP-Na_2_, 0.1 mM GTP, and 10 mM glucose (pH 7.2, adjusted with Tris base). Cells were superfused with standard external solution in the presence or absence of carbachol focally using a Y-tube perfusion system.

### Statistical Analysis

Results are presented as the mean ± s.e.m. from at least three independent experiments. Statistical comparisons were made using Student’s *t*-test (for two groups) or analysis of variance followed by the Student-Newman-Keuls procedure (for multiple groups). Values of P < 0.05 were considered significant.

## Additional Information

**How to cite this article**: Kitajima, N. *et al*. TRPC3 positively regulates reactive oxygen species driving maladaptive cardiac remodeling. *Sci. Rep.* **6**, 37001; doi: 10.1038/srep37001 (2016).

**Publisher’s note:** Springer Nature remains neutral with regard to jurisdictional claims in published maps and institutional affiliations.

## Supplementary Material

Supplementary Information

## Figures and Tables

**Table 1:** Cardiac parameters measured by Millar Catheter.

	TRPC3^(+/+)^ sham (n = 6)	TRPC3^(−/−)^ sham (n = 6)	TRPC3^(+/+)^ TAC (n = 13)	TRPC3^(−/−)^ TAC (n = 10)
Heart Rate (bpm)	410 ± 6	400 ± 4	393 ± 6	402 ± 3
LVESP (mmHg)	135 ± 1	151 ± 5	180 ± 10**	232 ± 11^##^
LVEDP (mmHg)	0.8 ± 0.5	1.4 ± 0.9	5.4 ± 0.7**	2.7 ± 1.1^#^
dP/dt max (mmHg/sec)	10503 ± 531	12288 ± 354	8552 ± 282*	12750 ± 802^##^
dP/dt min (mmHg/sec)	7298 ± 104	8201 ± 341	6598 ± 381	8122 ± 466
tau (msec)	12.5 ± 0.3	12.1 ± 0.4	16.0 ± 0.9*	13.5 ± 0.8^#^

HR, heart rate; LVESP, left ventricular end systolic pressure; LVEDP, left ventricular end diastolic pressure; dP/dt max, maximal rate of pressure development; dP/dt min, maximal rate of decay of pressure; tau, monoexponential time constant of relaxation.

Data are mean ± s.e.m. *P < 0.05, **P < 0.01 v.s. TRPC3 (+/+) sham, and ^#^P < 0.05, ^##^P < 0.01 vs. TRPC3 (+/+) TAC.

**Table 2:** Weight parameters.

	TRPC3^(+/+)^ sham (n = 6)	TRPC3^(−/−)^ sham (n = 6)	TRPC3^(+/+)^ TAC (n = 13)	TRPC3^(−/−)^ TAC (n = 10)
BW (g)	27.4 ± 0.8	29.5 ± 1.0	25.6 ± 0.6	27.6 ± 0.6
HW/TL (mg/g)	71.8 ± 1.4	74.6 ± 1.5	99.8 ± 5.5**	110.2 ± 3.5^#^
LunW/TL (mg/g)	80.5 ± 0.6	84.6 ± 1.8	89.4 ± 5.9	90.5 ± 2.3
LivW/TL (mg/g)	652.6 ± 33.7	600.1 ± 42.9	542.5 ± 19.6	591.1 ± 24.9
KidW/TL (mg/g)	95.9 ± 1.5	95.3 ± 2.0	94.8 ± 2.3	99.4 ± 3.8

HW/TL; heart weight per tibial length, LunW/TL; lung weight per tibial length, LivW/TL; liver weight per tibial length, KidW/TL; kidney weight per tibial length.

Data are mean ± s.e.m. **P < 0.01 vs TRPC3(+/+) sham, ^#^P < 0.05 vs TRPC3(−/−) TAC.
