# Supplementary material for: TRPC3 positively regulates reactive oxygen species driving maladaptive cardiac remodeling
Source: Sci Rep. 2016 Nov 11;6:37001. doi: 10.1038/srep37001 (PMC5105134; doi:10.1038/srep37001)
Supplement: Supplementary Information [file srep37001-s1.pdf]

TRPC3 positively regulates reactive oxygen species driving maladaptive cardiac remodeling

Naoyuki Kitajima, Takuro Numaga-Tomita, Masahiko Watanabe,

Takuya Kuroda, Akiyuki Nishimura, Kei Miyano, Satoshi Yasuda,

Koichiro Kuwahara, Yoji Sato, Tomomi Ide, Lutz Birnbaumer, Hideki Sumimoto,

Yasuo Mori and Motohiro Nishida

Supplementary Table 1 List of siRNAs

Target	Sequence (5'-3')
rat TRPC3 #1	AUAUCGUGUUGGCUGAUUGAGAAUG
rat TRPC3 #2	UCAUCUUCCUGGGUCUGCUUGUGUU
rat TRPC6 #1	UAAAUCUUGCAAUGAAUGAUGCUGC
rat TRPC6 #2	GGUCUUUAUGCAAUCGCGGUGGUUU
rat TRPC1	GGCGUGCGACAAGGGUGACUAUUAU
	Catalogue number (stealth RNAi)
rat NOX2 #1	RSS330363
rat NOX2 #2	RSS330364

Supplementary Table 2 List of Taqman probes and primer pairs for qRT-PCR

**Commercial Taqman probes**	**Taqman probe ID (Life technologies)**	
Mouse TRPC3	Mm00444690_m1	
Mouse TRPC6	Mm01176083_m1	
Mouse Cyba ($p22^{phox}$)	Mm00514478_m1	
Mouse Cybb (Nox2)	Mm00434757_m1	
Eukaryotic 18S rRNA		
**Primers for SYBR green**	**Sequence**	
rat TRPC1	Forward	ATCTTCATGTGCGGTCACAGT
	Reverse	TACATCTCAAGCCGCAAGCA
rat TRPC3	Forward	TCAATCAGCCAACACGATATCAGCA
	Reverse	TTCCTCCGTCGCTTGGCTCTTA
rat TRPC6	Forward	TGGCAAGTCCAGCATACCTG
	Reverse	CTCCGTGTTTCTGCAGAGGT
